# Impact of a Clinical Decision Support Alert on Informed Consent Documentation in the Neonatal Intensive Care Unit

**DOI:** 10.1097/pq9.0000000000000713

**Published:** 2024-02-05

**Authors:** Emily Sangillo, Neena Jube-Desai, Dina El-Metwally, Colleen Hughes Driscoll

**Affiliations:** From the Department of Pediatrics, University of Maryland School of Medicine, Baltimore, Md.

## Abstract

**Background:**

Informed consent is necessary to preserve patient autonomy and shared decision-making, yet compliant consent documentation is suboptimal in the intensive care unit (ICU). We aimed to increase compliance with bundled consent documentation, which provides consent for a predefined set of common procedures in the neonatal ICU from 0% to 50% over 1 year.

**Methods:**

We used the Plan-Do-Study-Act model for quality improvement. Interventions included education and performance awareness, delineation of the preferred consenting process, consent form revision, overlay tool creation, and clinical decision support (CDS) alert use within the electronic health record. Monthly audits categorized consent forms as missing, present but noncompliant, or compliant. We analyzed consent compliance on a run chart using standard run chart interpretation rules and obtained feedback on the CDS as a countermeasure.

**Results:**

We conducted 564 audits over 37 months. Overall, median consent compliance increased from 0% to 86.6%. Upon initiating the CDS alert, we observed the highest monthly compliance of 93.3%, followed by a decrease to 33.3% with an inadvertent discontinuation of the CDS. Compliance subsequently increased to 73.3% after the restoration of the alert. We created a consultant opt-out selection to address negative feedback associated with CDS. There were no missing consent forms within the last 7 months of monitoring.

**Conclusions:**

A multi-faceted approach led to sustained improvement in bundled consent documentation compliance in our neonatal intensive care unit, with the direct contribution of the CDS observed. A CDS intervention directed at the informed consenting process may similarly benefit other ICUs.

## INTRODUCTION

While healthcare organizations prioritize informed consent and provide well-defined governance, practices in documenting consent in the intensive care unit (ICU) are variable.^1–3^ Practice variations may be attributed to differing interpretations of “emergent intervention” justifying waiving consent, limited access to surrogate decision-makers, and time constraints in a fast-paced ICU setting.^1–4^ One study in an adult ICU reported that they performed almost half of their procedures without documented consent before establishing a universal consent process, which bundles consents for multiple procedures.^4^ In critically ill adults, bundled consent improves consent documentation compliance and results in higher family and nursing satisfaction with the quality of ICU care.^1,5,6^ One pediatric ICU successfully eliminated the performance of invasive procedures without guardian consent under “emergent circumstances” by utilizing bundled consent.^7^ Even when consenting occurs promptly, proper documentation of informed consent remains elusive. A report of one pediatric ICU indicated procedure consent documentation as high as 89%, with only 46% of those appropriately documented.^5^

In the neonatal intensive care unit (NICU), obtaining consent has additional unique challenges. These include the necessity for multiple procedures during a prolonged hospitalization and the limited availability of a proxy to consent due to factors like severe fatigue, extensive medical care needs, or lack of decision-making capacity. These challenges emphasize the significance of enhancing the NICU consenting process, including promoting the acquisition of consent for potential treatments upon admission. Our NICU developed a bundled consent encompassing authorization for emergent and routine procedures (eg, intubation, mechanical ventilation, lumbar puncture, thoracentesis, and placement of central lines, enteral tubes, and chest tubes). Unit leadership established the expectation for consent documentation to be completed within 24 hours of admission, in line with the hospital-wide goal, as many procedures are often performed during this timeframe. Before 2019, there was no auditing of bundled consent documentation compliance nor education on its use. However, an audit of 46 patients between December 2019 and March 2020 revealed that none had an appropriately documented bundled consent form, as the forms were either improperly completed or not utilized. Furthermore, most providers were unaware that a bundled consent document existed. Our goal was to increase compliance with the bundled consent form from 0% to 50% over 1 year.

## METHODS

### Setting and Patient Population

This quality improvement initiative occurred at a Level IV NICU in a large academic center that utilizes Epic (Epic Systems Corp, Verona, Wis.), a leading electronic health record (EHR) vendor. Our 52-bed NICU serves a high-risk birthing center and provides advanced subspecialty and surgical services to the neonatal population in Maryland, resulting in more than 800 annual admissions. Patients are cared for by attending physicians, advanced practice providers, fellows, and pediatric residents, who are responsible for obtaining bundled consent. Providers can access and print the NICU bundled consent form from our hospital’s electronic consent form library. Paper copies of the consent form are available in our NICU for internet downtimes. We assembled a multidisciplinary team dedicated to this initiative consisting of physicians, advanced practice providers, nurses, clinical informatics specialists, and EHR builders. The team used a Plan-Do-Study-Act model involving a literature review, stakeholder engagement, identification of key drivers, implementation of tailored interventions, and evaluation of their impact on consent documentation compliance.

### Data Collection

We collected data during monthly chart audits. As the primary auditors, clinicians from the multidisciplinary team aimed to perform a minimum of 15 monthly audits in accordance with a hospital-wide target. Each audit consisted of manually verifying the presence of bundled consent documentation and confirming all the required parts of the consent were appropriately addressed. Auditors determined if consents were “missing,” “present but non-compliant,” or “present and compliant.” Our institutional policies on the consenting process governed requirements for achieving compliance.

### Analysis

We analyzed monthly compliance on a run chart using standard run chart interpretation rules to calculate the center line (median).^6^ We analyzed changes in noncompliance of specific consent form elements compared to baseline data using a student’s *t* test.

#### Plan-Do-Study-Act Cycles:

**Fig. 1.**
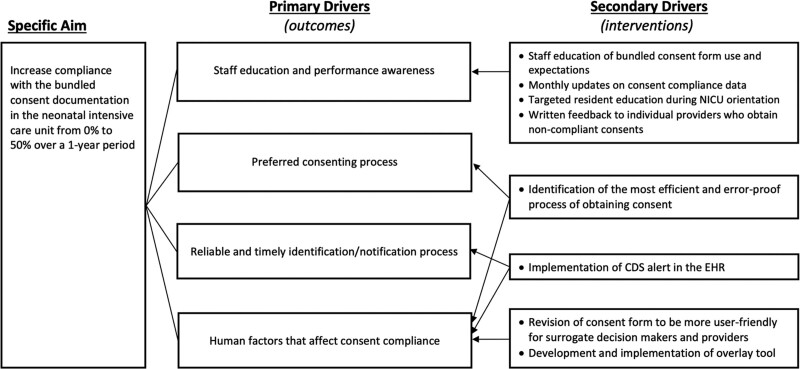
Key Driver Diagram for Increasing Bundled Consent Documentation Compliance in the NICU.

 We summarized factors that influenced implementation strategies for Plan-Do-Study-Act cycles in a key driver diagram (Fig. 1).

#### Cycle 1: Staff Education and Performance Awareness:

In December 2019, leaders of the quality improvement team introduced the bundled consent form and expectations for its use to consenting providers (attending physicians, advanced practice providers, fellows, and pediatric residents) and nursing. In March 2020, we started sharing data on consent compliance during monthly quality improvement meetings. These meetings provided information on areas of noncompliance and permitted users to give feedback on barriers to utilizing the bundled consent form. In August 2020, we performed additional education on noncompliance to neonatal providers as cultural encouragement. In October 2020, a hospital-wide mock Joint Commission survey was performed, further emphasizing consent compliance. In February 2021, targeted resident education during NICU orientation began. In March 2021, the Joint Commission survey occurred. We sent additional reminders to clinicians and nurses during that period to complete consent forms. From December 2021 to June 2022, we provided direct feedback via email to individual providers who obtained noncompliant consent.

#### Cycle 2: Delineation of the Preferred Consenting Process:

**Fig. 2.**
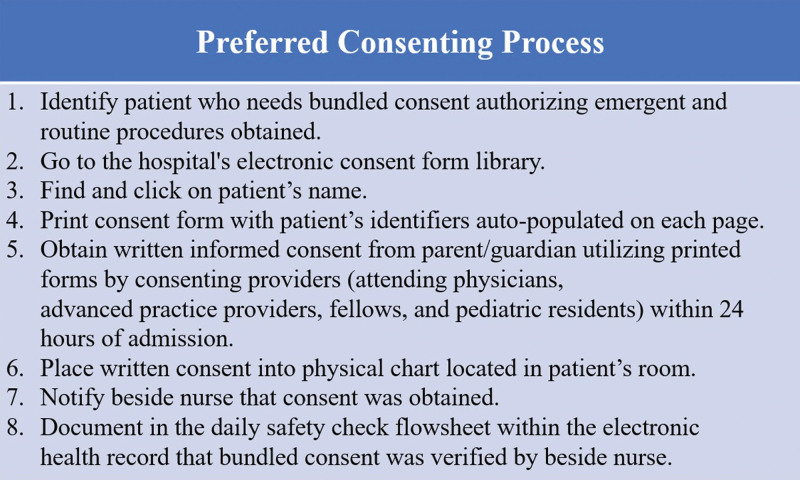
Preferred Order of Steps in the Consenting Process.

From March to April 2021, we observed an increase in noncompliant consent forms due to missing patient identifiers. This occurred because providers used pre-printed, blank consent forms placed in the bedside chart by unit secretaries as a courtesy instead of forms directly printed from the electronic library that autopopulate correct identifiers on every page. Once this deviation was identified, we educated clinicians, nursing, and unit secretaries on the preferred order of steps in the consenting process (Fig. 2), including discouraging the placement of blank forms in the physical chart.

#### Cycle 3: IT Integration Support:

**Fig. 3.**
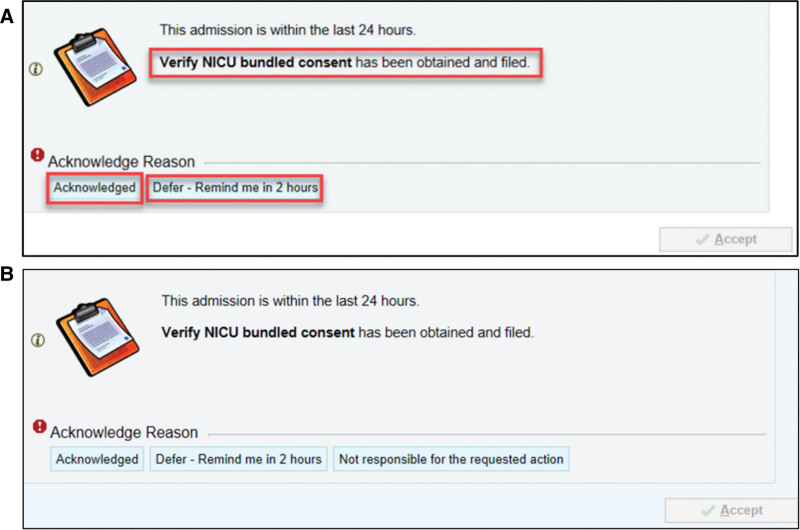
CDS to Obtain NICU Consent. A. Initial CDS developed and implemented. B. Revised CDS incorporating consultant feedback to include a third option to denote that this request was not their responsibility.

In September 2021, we launched a just-in-time interruptive clinical decision support (CDS) alert in the EHR to remind providers to obtain bundled consent. Focused on optimizing the judicious use of CDSs, the CDS governance committee guided the team to develop our CDS intervention, considering the five “rights” (the right information, to the right person(s), in the right intervention format, through the right channel, at the right time in the workflow).^8^ We summarized the rationale and the five “rights” framework for CDS development in Table. Following extensive discussion, the CDS governance committee determined that an active alert format, which interrupts the provider’s workflow and necessitates user interaction, was justified due to the baseline audit revealing zero percent compliance with bundled consent documentation and the substantial ethical and legal ramifications associated with nonadherence to the hospital’s informed consent policy. The CDS tool was programmed to check if the “Bundled Consent Verified” row in the daily safety check flowsheet was marked as completed in the first 24 hours of admission. If the consent documentation was not verified in the flowsheet, an initial interruptive CDS alert would fire a notification to any consenting provider who entered the patient chart, interrupting the user’s workflow. Team members receiving this alert could “acknowledge” the alert or “defer for 2 hours” (Fig. 3A). Selecting “acknowledged” indicated that the clinician was notified about the missing bundled consent and did not want another reminder in 2 hours. If bundled consent remained unverified in the flowsheet after 6 hours from the initial alert, a second CDS alert would trigger for that provider upon reentry. If a provider chose “defer,” the CDS intervention would send a second alert to that provider upon chart re-entry after two hours. Each provider selected the acknowledged reason individually, and their choice did not impact other providers accessing the chart. The CDS alert would not trigger to avoid alert fatigue if the nurse documented a consent form on file within the daily safety check flowsheet. The CDS alert also permitted an opportunity to provide direct feedback to the Information Technology (IT) team through the electronic alert itself. Each time an alert was delivered, the user was asked, “Was this advisory helpful?” Users could respond with a thumbs-up for positive feedback or a thumbs-down for negative feedback. Furthermore, a comment box was provided below the question for a detailed response.

**Table.** 5 “Rights” Framework for Developing a CDS Intervention Focused on Improving Bundled Consent Documentation Compliance.

CDS Right	Principle Feature
The right information	CDS intervention recommending timely confirmation of written bundled consent if consent was not documented verified within the daily safety check flowsheet in the first 24 hours of admission
To the right person(s)	CDS intervention directed to consenting providers only (eg, attending physicians, advanced practice providers, fellows, and pediatric residents)
In the right intervention format	CDS intervention formatted to be an active soft stop alert that interrupts the provider’s workflow and requires user interaction, increasing the chance of adherence to the CDS recommendation. Incorporating a soft stop nature to the alert allowed a provider to bypass recommendation with documentation of valid reasoning, providing flexibility to the user and limiting undesired disruptions in clinical workflow.
Through the right channel	CDS intervention delivered via EHR, an ideal platform for delivering an alert geared toward consenting providers
At the right time in the workflow	CDS intervention delivered between the first and twenty-fourth hour of patient admission, with the intent of obtaining consent on admission or early during NICU course. CDS firing was limited to the first 24 hours of admission due to the following reasons: (1) to comply with hospital-wide goal timing of obtaining consent, (2) to obtain timely consent before common procedures that are often performed during this timeframe, and (3) to reduce fatigue associated with repetitive firing alerts.

Abbreviations: CDS, clinical decision support; EHR, electronic health record; NICU, neonatal intensive care unit.

Due to programming limitations, we could not restrict the CDS alert to only those part of the NICU consenting process. As an unintended consequence, consultants also received the CDS alert. Due to nine instances of negative feedback from consultants on the irrelevance of the CDS intervention, we created a third option to select, stating, “I am not responsible for the requested action” (Fig. 3B). Selecting this option would prevent the alert from reappearing for that provider.

During the creation of the third selection in January 2022, the IT staff inadvertently programmed a row of logic in reverse, preventing the CDS alert from being delivered. We identified this error during a systematic review investigating an unusual drop in compliance (87% to 33%) from January 2022 to March 2022. The review revealed that several providers did not receive the CDS alert in recent months. In April 2022, EHR builders rectified the issue and reinstated the CDS alert.

#### Cycle 4: Consent Form Revision:

In October 2021, we revised the consent form to include more instructive statements. For example, in sections asking families to indicate their consent, we added directives such as “initial” to prompt the placement of the consenter’s initials on the form. Based on feedback from clinicians and nursing, we modified the order of procedures to start with more invasive and life-saving procedures (eg, thoracentesis), as providers felt those procedures required more alertness and attention from families during the consenting process than routine procedures (eg, nasogastric tube placement). As clinicians did not often cross out procedures that families declined, a “decline” option was added for each procedure to clarify whether a procedure was granted consent or declined (See figure, Supplementary Digital Content 1, which shows the example of consent revision to clarify documentation requirements. “*” denotes edited instructive elements targeting the 3 most common reasons for noncompliant bundled consents; “&” denotes revision to improve clarity on whether a procedure was granted consent or was declined; and “^” denotes additional instructive elements identified as a common cause of noncompliance. http://links.lww.com/PQ9/A537).

#### Cycle 5: Implementation of Overlay Tool:

In July 2022, we created an overlay tool using a clear acetate sheet to be placed atop the paper consent form. The tool featured red boxes to highlight noncompliant sections based on previous audit feedback. Two copies of the tool were placed in the team workroom, and consenting providers were educated on best using this during the consenting process.

### Ethical Considerations

The University of Maryland Baltimore institutional review board evaluated this quality improvement project and determined it was not human subjects research.

## RESULTS

**Fig. 4.**
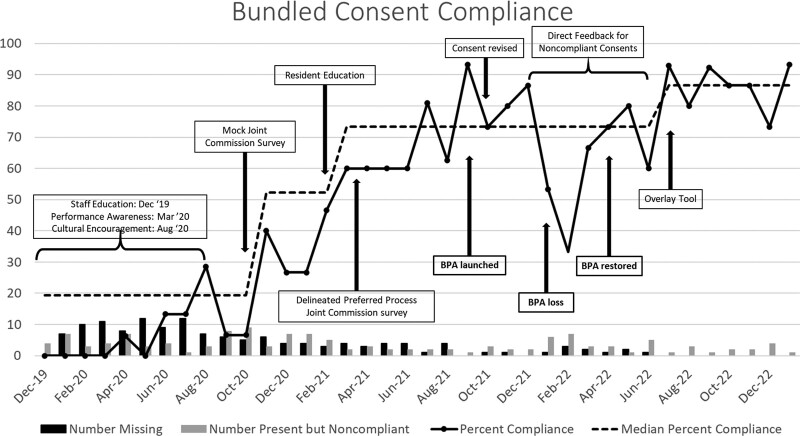
Bundled Consent Documentation Compliance Run Chart.

**Fig. 5.**
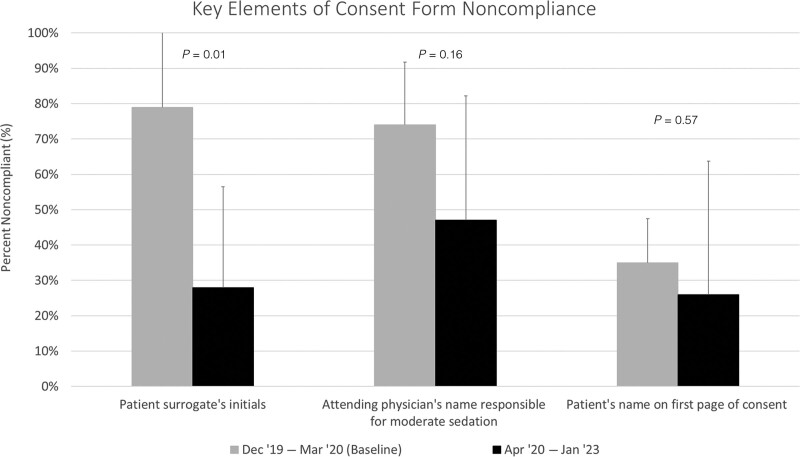
Most Common Deficiencies Contributing to Noncompliance Among Present but Noncompliant Forms.

We performed 546 audits out of 2584 admissions over 37 months, with an average of 14.8 monthly audits. The lowest monthly consent compliance was 0%, peaking at 93.3%. More consents were either missing or present but noncompliant in the first 18 months of the initiative, with an eventual absence of missing consents starting in July 2022 (Fig. 4). The three most common deficiencies in present but noncompliant consent forms were the lack of appropriate use of the patient surrogate’s initials to indicate procedure consent, missing documentation of the attending physician’s name responsible for moderate sedation, and the absence of the patient’s name on the first page of consent. The monthly noncompliance rate in each category decreased from April 2020 to January 2023 (Fig. 5) compared with baseline audit data. Median compliance rose from 0% to 86.6% over 37 months (Fig. 4). After the initiation of staff education and performance awareness strategies, consent compliance rose from 0% to a median of 19.3%. After disseminating the mock Joint Commission survey, a shift in compliance occurred, increasing median compliance to 52.3%. Median compliance further improved following resident education, the Joint Commission survey, and delineating the preferred consenting process. CDS implementation led to the highest monthly compliance in September 2021 of 93.3%. We observed an immediate decrease in compliance to as low as 33.3% in February 2022, following the inadvertent misprogramming of the CDS alert. Within one month of identifying the CDS error and reinstating proper logic programming, compliance improved to 73.3%. After restoring the CDS alert and introducing the overlay tool, median consent compliance shifted to its highest level. Negative feedback in response to the CDS alert decreased from nine instances between October and December 2021 to none from April to January 2023 after revising the CDS alert to include three response options (Fig. 3B).

## DISCUSSION

Despite the significance of adhering to the basic tenets of informed consent, there is limited literature on interventions to improve consent documentation in pediatrics, especially in addressing the distinct challenges in NICU documentation.^9^ These challenges notwithstanding, we used multiple Plan-Do-Study-Act cycles to promote the successful completion of the NICU admission bundled consent form. We found meaningful improvement in the rate of consent documentation after introducing staff education and performance awareness strategies, delineating the preferred consenting process, revising the consent forms, and creating an overlay tool. These interventions addressed barriers to awareness of expectations and time constraints while also considering human factors influencing consent compliance. Krvavac et al significantly improved consent documentation compliance using education and awareness interventions focused on socializing performance metrics. Furthermore, the authors found improper documentation of similar components of the consent form, such as the provider’s name. They surmised, and we have demonstrated that re-designing consent forms to be explicitly instructive reduces these areas of noncompliance.^10^

Our study is the first to illustrate improved consent compliance associated with implementing a CDS alert. Numerous studies have successfully implemented CDS interventions to improve pediatric healthcare delivery.^11–17^ Although the highest monthly consent compliance accompanied our CDS alert, its impact was unintentionally tested when the mis-programming of the CDS tool occurred. After we identified a sharp decline in consent documentation compliance and restored the logic programming, compliance returned concomitantly, highlighting the effectiveness of the CDS intervention as a strategy to improve adherence to recommendations. The success of the intervention is important because our CDS tool bypasses reliance on human factors. Unlike audit and feedback campaigns that may require repeated administration for sustained outcomes, a CDS alert provides continual computerized reminders, which has contributed to consistent success for ten months in our NICU.^12,18^

We partially attribute the success of our CDS intervention to the type of alert used. This interruptive alert provides timely and clinically relevant warnings and requires user response.^19,20^ Similar to our findings, Valvona et al demonstrated improved adherence to recommendations when using CDS interruptive alerts compared with passive alerts that do not require user interaction.^19^ Adherence to active alerts was over seven times higher than that observed for passive alerts when controlling for whether or not the alert was required.^19^ Other alert design factors, including character count, boxes, and activity links, can influence the impact of CDS tools and should be considered during implementation.

Concerns about the routine use of CDS alerts are documented in the literature. Many studies suggest CDS interventions are prone to malfunction, resulting in an EHR tool that does not work as designed or intended, as observed in our experience.^21–23^ Etiologies of CDS malfunction include but are not limited to build error, conceptualization error, defect in EHR software, and inadvertent enabling/disabling.^24^ Depending on the clinical circumstance, CDS malfunctions can potentially lead to patient safety risks and “alert fatigue.”^25–27^ As demonstrated in our experience and the literature, these potential risks underscore the necessity of having the appropriate IT infrastructure and effective governance to ensure the reliability of CDS systems.^21,24^ This requires personnel, training, technology, and funding to support successful CDS implementation.

Another well-described concern is the intrusiveness of CDS tools, potentially impacting the physician workflow.^19,27–29^ Several studies have identified and reported on best practices (eg, the 5 “Rights” of CDS and CDS governance structures) to avoid such issues and ensure successful CDS alert implementation.^19,25,27,30^ In our study, the CDS was programmed as a soft stop alert restricted to the first 24 hours of admission, allowing users to bypass recommendations with valid reasoning and limiting undesired disruptions in the clinical workflow. Because the standard of care involves waiving informed consent in emergent clinical scenarios, there were no delays in delivering care related to bundled consent documentation. Early in the CDS implementation, we received feedback that the alert was considered inappropriate and intrusive for consultants. Once we modified the CDS alert to incorporate user concerns, there was no negative feedback over 10 months.

There are several limitations to our study. We cannot assess role-specific contributions to performance because our data provided only information about provider types for present but noncompliant consents. Despite our study’s incomplete data on role contributions, existing literature suggests that trainees, including fellows (91%) and residents (66%), are primarily responsible for obtaining consent in the NICU and pediatric ICU.^31^ Considering this, our team focused on providing specialized education on bundled consent documentation to all trainees rotating in our unit. Similarly, we could not assess factors associated with the timing of consent completion (eg, the impact of weekend staffing on documentation compliance). Additionally, this is a single-center study involving one ICU, thus potentially limiting generalizability. However, specific interventions we implemented, such as the CDS tool, have improved a broad range of clinical workflows, lending itself to generalizability. Our study outcomes are limited to the presence of properly documented consent forms. They do not assess other important aspects of informed consent, such as the impact of improved consent documentation on families’ autonomy and parent/guardian understanding of their child’s illness and the risks/benefits of each procedure.^32^ However, a recent study involving one pediatric ICU demonstrated bundled consent utilization not only did not increase acute parental stress but also improved parental perception of their child’s severity of illness, supporting the ethical use and benefits of bundled consent.^7^ Lastly, we did not assess whether there was a reduction in the percentage of procedures performed without documented consent.

## CONCLUSIONS

This study used quality improvement methods to improve bundled consent documentation compliance in a single Level IV NICU. Implementing a CDS alert and its inadvertent discontinuation revealed a CDS intervention’s direct and positive impact on the NICU informed consent process. With the appropriate CDS governance and IT infrastructure in place to ensure reliability, this high-yield intervention may improve consent documentation compliance in a broader range of settings.

## Supplementary Material


