# Supplementary material for: Impact of a Clinical Decision Support Alert on Informed Consent Documentation in the Neonatal Intensive Care Unit
Source: Pediatr Qual Saf. 2024 Feb 5;9(1):e713. doi: 10.1097/pq9.0000000000000713 (PMC10843373; doi:10.1097/pq9.0000000000000713)
Supplement: Supplementary file 1 [file pqs-9-e713-s001.pdf]

________________________ * **(Patient name)**, has been admitted to the neonatal intensive care unit (NICU). During the course of care in the NICU some invasive procedures may need to be performed. These procedures are routinely performed in the NICU and are an integral part of the care needed. The doctors, nurse practitioners and nurses believe that the potential benefits of these procedures are greater than the potential risks of the procedures. The team is always available for further discussion and to answer any questions. Not all of the following procedures may be done but the procedures and potential risks associated with them may include:

Thoracentesis/Chest tube insertion: This involves the sterile placement of a needle (thoracentesis) or a tube (chest tube) through the chest wall between the ribs. This is done to remove air or fluid in the space around the lung, which allows the lung to re-expand. The needle is briefly inserted to evacuate air. A chest tube can stay in place longer for continued evacuation or air or fluid.

Possible risks include:

- Bleeding from the insertion site
- Infection at the insertion site and/or space around the lung
- Puncture of the lung. Puncture of solid organs in the chest or abdomen.
- Injury to breast tissue leading to absence or deformity of the breast
- Blood vessel and/or nerve injury.

Alternative: None

Initial to indicate selection: *

- Thoracentesis CONSENT________ or DECLINE________
- Chest Tube CONSENT________ or DECLINE________

Central lines: Inserting central lines involves placing a plastic tube (catheter), under sterile conditions, into one of the blood vessels. For the umbilical lines, the catheter is placed in one of the blood vessels in the umbilical cord. For the PICC line, the catheter is placed in one of the smaller veins, usually in the arms, legs, or scalp, and then threaded into a larger vein near the heart. An X-ray and/or an ultrasound is done to make sure the catheter is in the right place. For the peripheral arterial line (PAL), the catheter is placed in an artery, usually in the wrist, but sometimes in the groin, foot, or upper arm. We use the central lines to give IV fluids, nutrition, and medications, to monitor blood pressures, and to get blood samples for testing.

Possible risks from central lines:

- Bleeding around or through the catheter
- Infection at the site where the catheter enters in the skin
- Air entering in the veins or artery through the catheter
- Catheter breaking inside the vein or artery
- Catheter moving outside of the vein or artery
- Fluid leakage outside the catheter and around the lung or heart
- Air coming out of the lung and causing collapse of the lung (pneumothorax)
- Irregular heart rate (arrhythmia)
- Blood vessel and/or nerve injury
- Clot formation in the vein or artery

Alternative: Frequent needle sticks for arterial blood samples and/or replacement of peripheral IVs. Frequent blood pressure measurement using a cuff.

Initial to indicate selection:

- Umbilical vein catheterization (UVC) CONSENT________ or DECLINE________
- Umbilical artery catheterization (UAC) CONSENT________ or DECLINE________
- Peripherally inserted central catheter (PICC) CONSENT________ or DECLINE________
- Peripheral arterial line (PAL) CONSENT________ or DECLINE________

&

I consent for IV sedation (a medicine that makes your baby comfortable and relaxed, it is usually given through an intravenous line; a small tube that is placed in a vein in an arm or leg using a small needle) during the procedures or other medications that may be routinely advisable during the performance of these procedures. In addition there may be other procedures required that are not covered by this form.

This form authorizes and directs * ________________________ and the Neonatal Intensive Care team to
Attending Physician Name (required)
perform (if needed) the aforementioned procedures. I recognize that there may be other more remote risks or complications associated with these procedures that may arise.

Signature of patient or surrogate: ________________________ Date: __________ Time: __________
^ (Signature, Date and Time must be in patient or surrogate's own hand)

Relationship to patient, if surrogate: ________________________
^ (If patient is physically unable to sign, but able to consent, document informed consent using the "verbal" witness signature line)

Signature of healthcare provider: ________________________ Date: __________ Time: __________

Healthcare provider printed name or ID #: ________________________

☐ Healthcare provider is bilingual and provided language concordant care for consent.

☐ Interpreter used: Name or Interpreter # ________________________

Telephone Consent obtained by Provider *(see signature above)*

From ________________________ *(Print the name of the legal guardian or surrogate decision-maker),*

________________ *(print the relationship of the consenter to the patient).*

Telephone consent witnessed by: ________________________ *(Print name)*

Witness Signature: ________________________ Date: __________ Time: __________

Verbal consent witnessed by: ________________________ *(Print name)*

Witness Signature: ________________________ Date: __________ Time: __________

Example of consent revision to clarify documentation requirements. * denotes edited instructive elements targeting the 3 most common reasons for noncompliant bundled consents; & denotes revision to improve clarity on whether a procedure was granted consent or was declined; ^ denotes additional instructive elements identified as a common cause of noncompliance.
